# Relations between Concurrent Longitudinal Changes in Cognition, Depressive Symptoms, Self-Rated Health and Everyday Function in Normally Aging Octogenarians

**DOI:** 10.1371/journal.pone.0160742

**Published:** 2016-08-23

**Authors:** Elisabet Classon, Katarina Fällman, Ewa Wressle, Jan Marcusson

**Affiliations:** 1 Department of Acute Internal Medicine and Geriatrics, Linköping University, Linköping, Sweden; 2 Department of Clinical and Experimental Medicine, Linköping University, Linköping, Sweden; Nathan S Kline Institute, UNITED STATES

## Abstract

Ability to predict and prevent incipient functional decline in older adults may help prolong independence. Cognition is related to everyday function and easily administered, sensitive cognitive tests may help identify at-risk individuals. Factors like depressive symptoms and self-rated health are also associated with functional ability and may be as important as cognition. The purpose of this study was to investigate the relationship between concurrent longitudinal changes in cognition, depression, self-rated health and everyday function in a well-defined cohort of healthy 85 year olds that were followed-up at the age of 90 in the Elderly in Linköping Screening Assessment 85 study. Regression analyses were used to determine if cognitive decline as assessed by global (the Mini-Mental State Examination) and domain specific (the Cognitive Assessment Battery, CAB) cognitive tests predicted functional decline in the context of changes in depressive symptoms and self-rated health. Results showed deterioration in most variables and as many as 83% of these community-dwelling elders experienced functional difficulties at the age of 90. Slowing-down of processing speed as assessed by the Symbol Digits Modality Test (included in the CAB) accounted for 14% of the variance in functional decline. Worsening self-rated health accounted for an additional 6%, but no other variables reached significance. These results are discussed with an eye to possible preventive interventions that may prolong independence for the steadily growing number of normally aging old-old citizens.

## Introduction

Aging is the result of a lifetime of accumulated changes that eventually affect the capacity to live an autonomous life. Physical and mental health, nutritional status and lifestyle habits, but also age-related or pathological cognitive changes [1, 2] are known to affect instrumental abilities of daily living (IADLs). With the steady aging of the world’s population there has been a growing interest in interventions to prolong independence. Performance on cognitive tests tend to show subtle declines well before everyday functioning is affected [3] and could therefore be useful clinical predictors. It is however still not clear how declines in cognitive ability impact IADL in aging, alone or as compared to changes in general and mental health [2, 4, 5]. The primary aim of the present study is to examine if cognitive decline, global or domain-specific, predicts functional decline in a subgroup of well-defined and intellectually healthy 85 year olds that were followed during 5 years as part of the Elderly in Linköping Screening Assessment 85 (ELSA 85, [6]) study.

Reviewing the literature to date, Royall, Lauterbach [2] found that cognition accounted for around 20% of the total variance in everyday function across studies and elderly populations, but the differences between individual studies were large. The cognition-IADL relationship varies depending on which population of elderly individuals is examined, and so does the sensitivity of different assessment methods. For example, as much as 50% of the variance in IADL-ability has been attributed to cognition in the early stages of Alzheimer’s disease [7] but the relationship is typically much smaller in individuals with more preserved intellectual capacity [8–10]. A brief screening instrument like the Mini-Mental State Examination (MMSE, [11]), which takes only a few minutes to administer and gives a crude, global measure of cognitive impairment, may thus be a good predictor of IADL in individuals who are not cognitively healthy [12, 13]. More sensitive, domain-specific neuropsychological tests might however be needed for normally aging individuals [9, 14, 15]. For example, Marshall et al [9] found an association between the MMSE and functional impairment in individuals with Alzheimer’s disease, but not in individuals that were either cognitively healthy or had mild cognitive impairment (MCI). Instead, measures of cognitive speed and delayed recall were related to IADL in these groups.

The MMSE is however typically what is used in clinical practice [16] but because it lacks sensitivity and items assessing executive function [17] it is often complemented by various forms of short multi-domain screening [18]. The Cognitive Assessment Battery (CAB, [19]) is an example of a screening battery that goes beyond the MMSE but can be administered in 30 minutes by staff who has not undergone extensive training. The CAB is a compilation of short (or shortened forms of) established neuropsychological tests that are scored separately and assess function in five cognitive domains: verbal episodic memory, visuospatial function, speed and attention, language and executive function. The value of such intermediary forms of cognitive assessment in predicting functional outcome is less well studied, although in many cases they represent the maximum level of information available to clinicians in treatment planning. In this study, the MMSE and the CAB subtests are compared in terms of their relation to IADL.

A number of cross-sectional and, more recently, longitudinal studies have examined predictors of functional decline. While longitudinal studies solve many of the problems associated with cross-sectional designs, they typically collapse participants across wide age-ranges, e.g. 65 and above [15, 20–23], with considerably fewer old-old (85 years and upwards) than younger participants. There is however evidence that cognitive decline in normal aging initially proceeds at a slow rate and then accelerates at around the age of 75, the mean age in many aging studies. Indeed, many cognitive abilities have been found to be well-preserved prior to the mid-seventies [5, 24] and the onset of decline might well continue to be delayed. Steady improvements in intellectual integrity and emotional well-being has been found for successively later-born, but same-age, cohorts–a development that is thought to mirror the decisive environmental and sociocultural changes that have taken place in recent history, including better educational quality, health care and lifestyle habits [25, 26].

Further, the degree of decline tends to vary across different cognitive domains [5, 27], some of which are more important to independence than others [20, 22, 28]. The relation between cognition and everyday function may thus differ between for example 85 year olds and 65 year olds and whether it does or not may depend on the domain examined.

Performance on most cognitive tests is influenced by factors like premorbid intelligence, educational attainment and socioeconomic status through life [29]. These factors may be challenging to estimate, which in turn makes it difficult to judge if test performance mirror a deterioration or not [30]. Change in cognition can however be established by re-testing, which is often done during investigation of MCI or dementia. Importantly, when two variables evolve in parallel during aging it strongly suggests they are dependent on each other. Examining change over time rather than baseline performance thus adds knowledge about the evolution of impairments [31] as well as clinical applicability. Few studies [15, 20, 31] have so far examined how longitudinal change in specific cognitive domains relate to change in everyday function and, to our knowledge, none has done so using data from a well-defined age-cohort.

Many factors besides cognition influence everyday function in normal aging [1]. Problems with mobility and visual impairment may certainly lead to increasing difficulties in using public transportation irrespective of, for example, gradual memory loss. Depression has been associated not just with functional decline [32], but also with a steeper cognitive decent in many (see for example [33]) but not all [4] studies. Based symptoms may independently contribute as much as cognitive ability to everyday function in older, intellectually healthy individuals [8] and even subsyndromal depressive symptomatology, i.e. below the threshold for minor depression, impact IADL ability [32]. In turn, mental health is affected by sociocultural factors such as social support, physically active life-styles and the social status afforded to the elderly [34, 35]. Low self-rated health is another important predictor of functional decline in older adults [5, 36]. According to earlier cross-sectional studies, lower self-rated health was strongly associated with IADL difficulties in the larger ELSA 85 sample at baseline, but the association between self-rated health and general cognitive ability was small [37]. Further, both self-rated health and depressive symptoms were more strongly associated with IADL difficulties than global cognition [38]. Depressive mood and self-rated health tend to deteriorate with age but these changes are seldom included when examining relations between cognitive and functional decline. To our knowledge, no previous study has examined the contributions of longitudinal change in both depressive mood, self-rated health and cognition to IADL decline.

The main objective of the present study was to examine how change in cognition as measured by two screening instruments (the CAB and the MMSE) used in primary and secondary care relates to change in IADL in intellectually healthy octogenarians. Non-cognitive factors associated with IADL were also examined, particularly changes in self-rated health and presence of depressive symptoms. These data were analyzed to answer the following research questions:

1. How does cognition, IADL, depressive symptom and self-rated health evolve in this cohort of normally aging old-old individuals?
2. Does concurrent change in cognition, depression and self-rated health predict IADL decline in this group?
3. Is any relation between decline in cognition and IADL best captured by the MMSE or by domain specific measures from a short screening battery?

Based on previous literature, declines in all variables were hypothesized [5, 39]. Cognitive change in domain specific measures, particularly in those targeting executive functions and processing speed, were expected to be better predictors of functional decline than the MMSE in these intellectually well-preserved individuals [9, 14, 15, 40]. Because earlier studies have found relationships between depressive symptoms or self-rated health on the one hand, and functional decline on the other, a similar pattern was expected in the present study [8, 32, 37, 38]. However, late-onset depressive symptoms are often related to incipient MCI or dementia [41], conditions that were carefully excluded in the present study. Further, longitudinal studies have found that self-rated health may remain relatively stable over time, in spite of worsening physical health [42, 43]. Thus, cognitive change was expected to be a stronger predictor of functional decline than changes in mental or general self-rated health.

## Materials and Methods

The ELSA 85 [6] is a longitudinal population study that has followed a cohort initially assessed at the age of 85 in 2007 (TI). Two follow-ups have been completed to date, one after 1 year (T2), and one after 5 years at age 90 (T3). T1 and T2 consisted of 3 phases: a postal questionnaire, a home visit by an occupational therapist and a visit to the Memory clinic in Linköping. The MMSE and self-report questionnaires concerning functional status were administered during the home visit. The visit to the clinic included administration of the CAB followed by medical examination and history taking. The protocol at T3 was for various reasons shortened to include only a home visit during which the MMSE, the CAB and self-report questionnaires were administered. Only data from T1 and T3 are analyzed in the present study. The instruments used that are relevant to the present study are described in detail below (see Measurements). The ELSA 85 study, including permission to obtain data from all registers held by the County Council of Östergötland, has been approved by the Research Ethics Committee of Linköping University, Sweden (2006: 141-06, 2012: 332-31). Written informed consent was collected by all participants at each step of the study.

### Participants

All 650 residents in the municipality of Linköping born in 1922 were invited to participate during the course of 2007 (T1). In total, 338 individuals participated in all 3 phases of T1 and 113 of these were willing and able to also participate at T3. From this group of 113 individuals, those with a documented or self-reported history of diseases known to affect cognition were identified and excluded. Medical records were systematically scrutinized at both T1 and T3. Specific exclusion criteria were a history of neurological (e.g. stroke, Parkinson’s disease, epilepsy, Huntingdon’s disease, polio), cognitive (e.g. dementia, MCI, confusion), or psychiatric (e.g. psychosis) disease and/or drug abuse. Participants with diagnosed depression were included if they were on stable medication and scored below the cutoff for current depressive symptomatology on a self-report questionnaire (i.e. a GDS-15 score < 6, see Measurements for a detailed description of the instrument) at baseline. An additional exclusion criteria was MMSE performance below the 16th percentile based on norms adjusted for age and education level [44]. Following this procedure, 30 individuals (27%) were excluded. The remaining 83 all lived independently throughout the study. Demographic characteristics of this sample are detailed in Table 1.

**Table 1:** Demographic characteristics of the participants at inclusion (age 85).

	N (%)
Study participants	83 (100)
Gender	
Females	43 (52)
Males	40 (48)
Years in formal education	
5–8	51 (62)
9–12	14 (17)
13-	15 (18)
Living alone	38 (46)
Friends close by	79 (95)
Visual impairment	65 (78)
Hearing impairment	59 (71)
Tobacco use	3 (4)
Alcohol consumption	
Never	14 (17)
Seldom	55 (66)
Regularly	14 (17)
Regular exercise	
30 min. once per week or less	14 (17)
30 min. twice per week or more	68 (83)
Comorbidity (>1 chronic disease)	50 (60)
Weight	
Underweight (BMI < 18,5)	1 (1)
Normal (BMI 18,5–24,9)	34 (41)
Overweight (BMI >24,9)	48 (58)

### Measurements

Cognitive functioning was assessed using the Mini-Mental State Examination (MMSE, [11]) and the Cognitive Assessment Battery (CAB, [19]). The MMSE consists of 12 items to assess orientation to time and place, attention, memory, language and visual construction. It yields a single total score ranging from 0–30 with lower scores denoting more impaired cognition.

The CAB consists of 10 subtests, detailed in Table 2. Episodic memory is assessed by a story recall test and speed of information processing by the Symbol Digits Modalities Test (SDMT, [45]) and the Trail Making Test part A (TMT-A, [46]). Short versions of the Boston Naming Test (BNT, [47]) and the Token test [48] are used to assess the language functions of naming and understanding. Visuospatial abilities are covered by a clock drawing task (CLOX, [49]), copying of a cube and a simplified version of the Rey Complex Figure (RCF, [50]). These are all visuo-constructive tasks scored in a similar way. In order to reduce the number of variables they were summed to create a composite score. Finally, the CAB includes two tests of executive function: the Victoria version of the Stroop test [51] to assess inhibition and the Parallell Serial Operations test (PaSMO, [52]) to assess mental control and divided attention.

**Table 2:** Description of the Cognitive Assessment Battery (CAB) subtests.

Domain/subtest	Task	Score	Range
Episodic memory			
- Story recall, immediate	Listen to a story and recall it verbatim	No. of segments recalled	0–21
- Story recall, delayed	Recall the story verbatim	‘‘	‘‘
Processing speed			
- SDMT	Match digits to symbols according to a key	No. correct responses in 90 s.	0–110
- TMT-A	Connect numbers in numerical order	Time in s.	-
Language			
- BNT, 30 items	Picture naming	No. of correct responses	0–30
- Token test, 6 items	Manipulate tokens according to spoken instructions	No. of correct responses	0–6
Visuospatial ability			
- CLOX	Draw and copy a clock, set to a specified time	No. of details correctly drawn	0–10
- Draw a cube	Copy a drawing of a cube	‘‘	0–2
- RCF, simplified version	Copy an abstract figure	‘‘	0–18
Executive function			
- Stroop test, Victoria version	Name the colors color-words are printed in	Time in s.	-
- PaSMO	Switch between saying letters and digits in alphabetical and numerical order	‘‘	-

IADL was assessed by the Instrumental Activity Measure (IAM, [53]), a self-report questionnaire covering perceived difficulty in the performance of 8 activities: locomotion outdoors, preparing a simple meal, cooking, using public transportation, small-scale shopping, large-scale shopping, cleaning and washing. There is one item per activity and four scoring alternatives: 1, “too difficult”; 2, “great difficulties”; 3, “some difficulties”, and 4, “no difficulties”. Thus, the lower the score, the larger the difficulty in performing the task. Ratings in each of the 8 domains were summed to yield a total score (IAMtot) spanning from 8 to 32.

The EQ-5D s is a self—report inventory designed to measure health outcome [54]. It includes a visual analog scale (EQ-VAS) that was here used to assess self-rated health. The scale runs from 100, “Best imaginable health state”, to 0, “Worst imaginable health state”.

Depressive symptoms were assessed by the GDS-15 [55], a shortened version of the geriatric depression scale (GDS, [56]). The instrument has a yes/no response format with a total score of 15 points. Scores of 6 or above indicates possible depression [57, 58].

The Timed Up & Go test (TUG, [59]) measures lower limb mobility. Participants are required to rise from an armchair, walk 3 meters, return to the chair and sit down. Dependent measure is the time in seconds to complete the task. This test was only performed at T1.

A range of demographic data was collected and processed for the present study: body mass index, alcohol consumption (users/non-users), smoking (yes/no), comorbidity (presence of ≥ two diseases or not), self-reported visual impairment (yes/no), habits of physical exercise (30 min walks at least a few times/week or other regular exercise/30 min walks once a week or no regular exercise) and social network (friends nearby/no friends).

### Statistical analyses

Both the CAB and the MMSE are intended for detection of cognitive impairment. Thus, when used in a cognitively healthy population there are ceiling effects and raw data are not necessarily normally distributed. The Wilcoxon signed ranks test was therefore chosen for pairwise comparisons. Change scores were computed by subtracting performance at T3 from performance at T1, or vice versa, such that deterioration (e.g. more depressive symptoms, slowed responses in timed cognitive subtests) is indicated by negative values and improvement by positive values. Correlations between change scores were examined using the Pearson correlation coefficient. Multiple linear regression analyses, using forced entry, were used to examine predictors of functional change. In the first step, all predictors were entered in one block according to their effect sizes. In step two, predictors were entered one by one in order of their standardized beta coefficients (βs) in the previous analysis as long as they contributed to the model (i.e. significant ΔR^2^ increase). Multicollinearity was checked by examination of intercorrelations, tolerances and variance inflation factors (VIF). The models were also examined by visual inspection of the distributions and normal probability plots of their standardized residuals.

## Results

### Cross-sectional at T1

**Fig 1:**
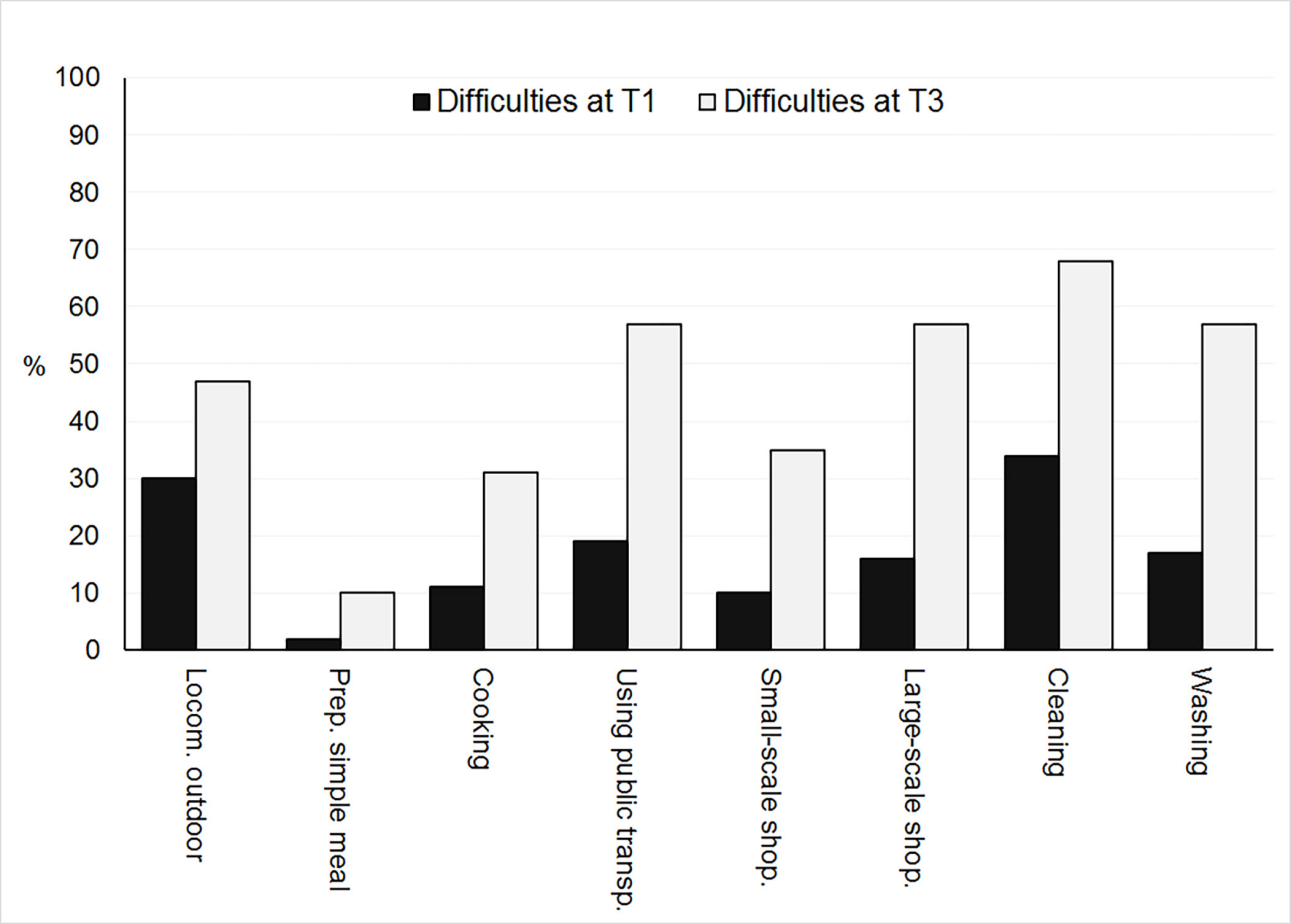
Diagram showing the percentage of participants reporting difficulties at T1 (black bars) and T3 (grey bars) for each of the IADL activities.

Descriptive data are presented in Table 3. At T1, median scores in both the MMSE and the CAB subtests were well within the range of normal performance according to the recommended MMSE cutoff score for cognitive impairment at ≥ 23 [60] and the CAB norms [19], excepting the Token test and the visuospatial ability score. According to the manual, full scores are expected in these two measures irrespective of age, something the present data suggest should be adjusted. With respect to functional ability, 49% reported difficulties in one or more IADL area (see Fig 1). Median self-rated health was somewhat higher than the population mean (72) for Swedes aged 75 or above [61] and few depressive symptoms were reported.

**Table 3:** Descriptive data at T1 and T3, p-values and effect sizes. The last two columns report the mean and SD of change scores (∆, computed by subtracting performance at T3 from performance at T1, or vice versa, such that deterioration is indicated by negative values) over the 5-year span of the study.

	T1, age 85		T2, age 90				Change (∆)	
	Mdn	*Range*	Mdn	*Range*	p*	Effect size (*r*)	M	*SD*
IADL (IAM tot)	32	*17*	27	*20*	**0.000**	0.63	-4.2	*5*.*8*
HrQoli (EQ-VAS)	75	*75*	67	*90*	**0.000**	0.43	-8.8	*18*.*6*
Depression (GDS-15)	1	*6*	2	*11*	**0.000**	0.56	-1.3	*2*.*0*
Mobility (TUG)	13	*40*	-	*-*	**-**	-	-	*-*
Global cognition (MMSE)	29	*6*	28	*9*	**0.000**	0.45	-0.9	*1*.*8*
CAB								
Episodic memory								
Story recall, immediate	6	*16*	6	*15*	0.088	0.20	-0.6	*2*.*9*
Story recall, delayed	7	*19*	6	*17*	**0.048**	0.23	-0.8	*3*.*2*
Processing speed								
SDMT	27	*39*	24	*40*	**0.000**	0.63	-3.8	*5*.*1*
TMT A	56	*225*	64	*88*	**0.000**	0.45	-10.3	*20*.*9*
Language								
BNT	26	*19*	25	*19*	**0.000**	0.48	-1.3	*2*.*5*
Token test	5	*5*	6	*5*	0.297	0.13	0.1	*1*.*0*
Visuospatial ability								
CLOX + Draw a cube + RCF	29	*13*	27	*24*	**0.000**	0.47	-1.6	*3*.*1*
Executive function								
Stroop	40	*80*	48	*127*	**0.003**	0.35	-5.8	*19*.*7*
PaSMO	87	*215*	92	*211*	**0.007**	0.36	- 17.1	*43*.*8*

* Wilcoxon signed ranks test (two-tailed) was used for all variables.

### Longitudinal, T1 to T3

#### Descriptive data

There were declines in most measures over the 5-year span of the study (Table 3). The median MMSE score was however still as high as 28 at 90 years of age. Median scores in the CAB subtests remained within normal ranges (apart from visuospatial ability as discussed above) but significant declines with age were found for the majority. The exceptions were language understanding (Token test), which remained intact, and immediate Story recall that however showed a tendency to deteriorate. The effect sizes displayed in Table 3 show that the largest effect of age was found for processing speed (SDMT, 0.63). As shown in Fig 1, perceived IADL difficulties also increased with as many as 83% reporting difficulties in at least one area at age 90. The differences between ratings at age 85 and age 90 were significant for all activities (*Z*s ≥ 2.45, *p*s ≤ .015), except preparing a simple meal (*Z* = 1.94, *ns*). Among the remaining activities, the largest effect sizes were found for cleaning (0.55), large-scale shopping (0.54) and using public transportation (0.50). Self-rated health (EQ-VAS) worsened over the 5-year span, as did depressive symptoms. At age 90, 7 participants (9%) had GDS-15 scores above 5.

#### Associations between change in cognition, depressive symptoms, self-rated health and functional ability

Because they were not sensitive to aging, the variables language understanding (Token test) and ability to prepare a simple meal were excluded from further analyses. Thus, a new IADL composite score was computed. Intercorrelations between all change scores are displayed in Table 4. Two initial multiple regression analyses, using the enter method, were conducted to examine the impact of 1) *background factors* (sex, years in education, lower limb function as measured by the TUG test, comorbidity, body mass index, alcohol consumption, smoking, visual impairment, habits of physical activity and social network) and 2) *baseline* cognition (MMSE and the CAB measures), depression and self-rated health, on IADL decline. This was done to ensure that important predictors were not missed in the subsequent analyses of *change* scores. Results showed that none of the variables reached significance as predictors (background factors: *β*s ≤ 0.18, *p*s ≥ .12; baseline measures: *β*s ≤ 0.30, *p*s ≥ .08).

**Table 4:** Pearson coefficients showing the interrelations between changes in cognition, depressive symptoms, self-rated health and IADL.

	Variable	1	2	3	4	5	6	7	8	9	10	11
1	∆IAM	-										
2	∆EQ-VAS	0,19										
3	∆GDS-15	0,18	0,36**	-								
4	∆MMSE	0,22*	0,07	0,20	-							
5	∆Story recall, immediate	0,19	0,09	0,13	0,19	-						
6	∆Story recall, delayed	0,15	0,01	0,07	-0,01	0,61**	-					
7	∆BNT	0,16	-0,15	0,04	0,00	0,14	0,02	-				
8	∆Visuospatial ability	0,21	-0,02	0,16	-0,16	0,18	0,20	0,28*	-			
9	∆TMT A	0,22	-0,05	0,12	0,11	0,04	0,15	0,04	0,19	-		
10	∆SDMT	0,38**	-0,14	0,14	0,07	0,09	0,25*	0,23	0,36**	0,32**	-	
11	∆Stroop	0,09	0,13	0,04	0,01	-0,11	0,06	-0,15	0,42**	0,35**	0,26*	-
12	∆PaSMO	0,14	0,02	-0,07	-0,11	-0,26	-0,14	0,03	0,13	0,21	0,21	0,25

*p < .05,

** *p* < .01.

The next set of multiple linear regressions, again using the enter method, was conducted to examine whether changes in self-rated health, depressive symptoms, global cognition or any of the CAB scores could predict functional decline. In a first exploratory analysis, all predictors were entered in one block arranged by their effect sizes (from largest to smallest). As indicated by the standardized regression coefficients, IADL decline was most strongly influenced by: 1) decline in processing speed (∆SDMT), 2) self-rated health (∆EQ-VAS), and 3) global cognition (∆MMSE; *β*s = 0.31, 0.25 and 0.19, respectively). When these predictors were entered one by one in a next set of regressions, results showed that decline in processing speed explained 14% of the variance in IADL decline (Table 5, Model 1). Adding change in self-rated health increased the amount of explained variance with 6%, but change in global cognition did not improve the model (Table 5, Models 2 and 3). Two follow-up regressions confirmed that change in processing speed accounted for an additional 11% and 19%, respectively, of the variance in IADL decline after the impact of either all the background factors, or baseline cognition, depression and self-rated health, were accounted for (*∆R*^*2*^ = 0.11, *F* = 7.92, *p* = .007; *∆R*^*2*^ = 0.19, *F* = 14.22, *p* = .000).

**Table 5:** The results of multiple linear regressions (enter method) with change in IADL as criterion variable.

Model		B	SE B	β	R^2^	ΔR^2^
1	Constant	-2.64	.81			
	∆SDMT	.42	.13	.38**	.14	.14**
2	Constant	-1.86	.87			
	∆SDMT	.46	.13	.41**		
	∆EQ-VAS	.07	.03	.24*	.20	.06*
3	Constant	-1,44	,89			
	∆SDMT	0,44	,12	,40**		
	∆EQ-VAS	0,07	,03	,23		
	∆MMSE	0,56	,34	,18	.23	.03

*p < .05,

** *p* < .01.

## Discussion

This longitudinal cohort study examined associations between changes in cognition, depression, self-rated health and IADL decline in normally aging octogenarians. Cognition was assessed by a global measure (the MMSE) and a more extensive screening battery with subtests assessing function in five separate cognitive domains (the CAB). The aims were to examine 1) longitudinal change in cognition, depressive symptoms, self-rated health and IADL, 2) if change in cognition, depressive symptoms and/or self-rated health could predict IADL decline and 3) if change in global, or domain-specific, cognitive screening performance accounted for more of the variance in IADL decline. Declines with age were expected for all variables and changes in both depression, self-rated health and cognition were hypothesized to be associated with functional decline. Specific measures of executive functions and processing speed were expected to be the best predictors.

The results did show significant deterioration in most measures during the 5 years the participants were followed, but only slowed processing speed and worsening self-rated health predicted functional decline.

### Longitudinal change

The pattern of cognitive change was largely in accordance with previous findings. Immediate memory and language understanding remained intact even to this advanced age [29, 62]. By contrast, global cognition, processing speed, naming, visuospatial ability and executive functions declined with effect sizes in the median-to-large range. Episodic memory decline was however small, contradicting the popular notion that old people have bad memory. Other longitudinal studies of normal aging have shown similar results [5, 20, 31, 63] indicating that, when spared from disease processes such as stroke or dementia [64], today’s elderly have well-preserved memory function. In line with theories proposing generalized slowing as the primary mechanism of age-related cognitive change [40, 65], processing speed showed the largest decline.

As for IADL ability, half of the participants experienced at least some difficulties in IADL-performance at the age of 85, a proportion that had risen to 83% by the end of the study. Thus, in this healthy community-dwelling sample, the vast majority experienced difficulties in performing everyday tasks at the age of 90. Indeed, problems using public transportation and cleaning were close to twice as common, already at age 85, as has been previously found for independently living 80–89 year olds in Sweden and the UK [66]. This is of note in view of the evidence that preserved functional ability is highly important to mental health and life satisfaction in old age [34]. Efforts should be made to investigate whether state and community policies aiming to facilitate independent living and travel may reduce such differences.

Frequency of depressive symptoms increased significantly during the study, which is in line with earlier findings of more depressive symptomatology in 90 year olds than in 85 year olds [39, 67]. Even so ratings were low, relatively few (9%) had GDS-15 scores indicating possible depression at age 90 and the majority either improved, remained stable or had but 1 more symptom than at age 85. Indeed, scores were comparable to the ones reported from 80+ inhabitants of Ikaria Island, an area known for its high number of very old and healthy inhabitants [68]. Put into perspective, it has been noted that although depressive symptoms tend to increase in old age, they do not reach the levels found in young adults. Counteracting factors such as life-long increases in emotion regulation skills is likey to be one, out of several, explanation [69, 70].

Self-rated health also deteriorated significantly for the participants as a group, but again, in as high a proportion as 41% it either remained stable or improved. High and stable self-ratings of health, in spite of increasing numbers of chronic diseases and waivering functional ability, is a well-known paradox in studies of aging, even found in centenarians [34]. While this phenomenon is likely to reflect processes such as adjustment of expectations [71], specifics of the self-report measures used are also important. For example, substantially more older individuals report declines when explicitly asked to rate how health has changed over time, rather than to rate the experience of health at the moment, as in the present study [42, 72].

### Predictors of IADL decline

Changes in global cognition (MMSE) did not predict functional worsening. This is consistent with findings from other longitudinal studies [14, 15, 20, 23] and show that the MMSE does not capture the early changes in cognition that affect everyday activities in normal aging. The same was true for most of the CAB subtests. Change in processing speed as measured by the SDMT however robustly explained 14% of the variance in IADL decline (for comparison, Royall, Lauterbach [2] found that cognitive variables explained a median of 15.9% of IADL variance across a number of studies). There was no association between change in executive function or number of depressive symptoms and increasing IADL difficulties, but worsening self-rated health predicted a small but significant portion of the functional decline.

Symbol substitution tasks like the SDMT are used as measures of processing speed [23, 73] or complex attention [29] and executive function [9, 20, 21], indicating the range of interrelated abilities they engage. Slowed processing speed has however been found to account for most of the age-related effects in these tasks [74, 75]. Performance depends on efficient transfer of information in frontoparietal networks involved in attention and working memory [76] and is sensitive to white matter damage in aging [77]. Low baseline performance has been associated with increased risk of mortality and/or functional decline in healthy older adults in longitudinal [23, 78] and cross-sectional studies [9, 73]. Only one study has, to our knowledge, included symbol substitution in a design examining concurrent trajectories of cognition and IADL in aging [20]. That study found decline in a multi-item executive functions assessment and speed as assessed by the TMT-A to be more closely related to functional decline. General effects of speed, attention and mental control are likely to be reflected by all of these instruments. For example, slowed processing speed limits higher cognitive abilities and explains part of the individual variance in executive function [79, 80]. Importantly, the links between white matter damage, reduced processing speed and functional decline, evident also in elderly without cognitive impairment, emphasize the relevance of assessing speed. Cognitive slowing may be an early marker of many dementing illnesses [29] and a potential indicator for treatment of cardiovascular risk factors [77], which in turn may reduce risk of dementia [81]. Further, training of processing speed in older adults has shown promising results, including long-term transfer effects to everyday abilities [82, 83]. Nevertheless, items assessing processing speed are absent from many cognitive screening instruments.

Results of a recent cross-sectional study [8] suggested that depressive symptoms are more strongly related to everyday function than cognition in older persons who are cognitively healthy. In the present study too, there were cross-sectional associations of moderat strength between depression and IADL at T1 and T2 (not reported). Contrary to expectation, change in depression over time was however not related to concurrent change in everyday function. Thus, the effects of depression on daily function may be limited to the short-term. Similar results have been reported for the association between depression and cognition. A recent longitudinal study of community-dwelling 70+ participants that spanned 12 years found no relationships between concurrent changes in depression and cognition when individuals with suspected cognitive impairment (MMSE<24) were excluded [4]. Even the baseline associations between depression and cognition disappeared. The authors concluded that undetected preclinical dementia may be responsible for part of the depression-cognition link often found in aging. If so, depression should also be less related to IADL ability when cognitive disease is absent, as suggested by the present study. Alternatively, depressive symptoms influence some activities more than others. For example, de Paula, Diniz [84], in a study including participants with normal cognition as well as MCI and mild dementia, found that depression did not predict variance in composite IADL scores, but impacted IADL activities specifically involving social contact. Such activities were not included in the present study, but should be considered in future works.

Instead, worsening self-rated health predicted a small, but significant, portion of the decline in functional ability in the present study. This provides further evidence for the well-known sensitivity of this simple measure. As discussed by Jylhä [72], it so comprehensive and non-specific that it is difficult to interpret. It is influenced by a range of psychological and cultural factors [85], but also varies systematically with objective health measures [43]. While the measure is powerful on it’s own, future studies should consider complementing it with a rating of perceived change in health [42, 72].

### Strengths and limitations

The longitudinal cohort design and the wide range of measures are strengths of the present work. The small sample size is however a limitation, related to the scarcity of 90 year olds that are both cognitively intact and physically able to participate. The use of short neuropsychological tests that can be administered by all professionals in primary or secondary care enhances applicability. The choice to analyze and report raw, rather than standardized, scores was also intended to increase clinical usefulness (i.e. when administered and analyzed as in a clinical setting, what does the test results and self-ratings indicate about everyday function). On the other hand, the range of scores is more limited in shortened test versions, with ceiling effects in many cases. Further, because of psychometric differences between the CAB subtests, conclusions about the relative contributions of underlying cognitive domains to IADL must be tentative even if the results are in line with findings from other longitudinal studies. Reliance on self-reports for assessment of health and IADL is not unproblematic [42, 86–88]. Lack of insight is less of a problem with cognitively intact participants, but factors such as social desirability or whether questions are phrased to elicit e.g. peer comparisons rather than perception of intra-individual change over time may influence results. Finally, the term “normal aging” refers to an absence of disease-related cognitive decline, something that cannot be guaranteed in spite of rigorous exclusion criteria.

## Conclusions

This study adds to the literature by investigating the interrelations between concurrent changes in multiple cognitive, health and functional variables in the same group of old-old and cognitively well-preserved individuals, followed over an extended period of time. To our knowledge this has not been done before. Results confirm that intra-individual declines in cognition, mood and self-rated health are present, but perhaps surprisingly modest, in octogenarians unaffected by cognitive disease. An overwhelming majority had functional difficulties at the age of 90, particularly in areas such as large-scale shopping, cleaning and using public transportation. Because loss of independence in turn increase the risk of other negative developments, long-term social policies aiming to improve, for example, the accessibility of public transportation may be imperative to support healthy aging. Contrary to expectations, change in depressive symptoms did not predict functional decline. This strengthens the theory that part of the relation seen in some, but not all, earlier studies between depression and functional ability in aging is related to incipient cognitive disease. Self-rated health was a better predictor, but still marginal. Objectively measured decline in mental processing speed was the best predictor of functional change. While this is in accordance with previous findings, assessment of processing speed is still rare in primary care. Cognitive slowing is related to cardiovascular disease, for which a number of medical and life-style interventions have proven effective and there is even some evidence that cognitive training may be helpful. The results of this study thus points to a number of preventive interventions that may prolong independence for the steadily growing number of normally aging old-old citizens.

## Supporting Information

S1 File This file provides the minimal data set used for the analyses presented in this article. (XLSX)
